# Seven-year follow-up of posterior chamber phakic intraocular lens with central port design

**DOI:** 10.1186/s40662-021-00247-1

**Published:** 2021-06-11

**Authors:** Luis Fernández-Vega-Cueto, Belén Alfonso-Bartolozzi, Carlos Lisa, David Madrid-Costa, José F. Alfonso

**Affiliations:** 1 Fernández-Vega Ophthalmological Institute, Avda. Dres. Fernández-Vega 114, 33012 Oviedo, Spain; 2 Optometry and Vision Department, Faculty of Optics and Optometry, University Complutense de Madrid, Madrid, Spain

**Keywords:** Implantable Collamer Lens, ICL, Myopia, Long-term

## Abstract

**Background:**

To assess the clinical outcomes of the Visian Implantable Collamer Lens (ICL) with a central port throughout 7 years of follow-up.

**Methods:**

Eighty-four eyes of 52 patients were evaluated over a follow-up period of 7 years after V4c ICL implantation. Uncorrected (UDVA) and corrected (CDVA) distance visual acuities, refraction, intraocular pressure (IOP), endothelial cell density (ECD) and vault were analysed.

**Results:**

The mean postoperative UDVA (logMAR) was 0.04 ± 0.11, 0.13 ± 0.19 and 0.17 ± 0.23 at 1-, 5- and 7-years, respectively (*P* < 0.0001). The mean CDVA (logMAR) remains unchanged throughout a 7-year follow-up period (0.02 ± 0.08 and 0.02 ± 0.08, at 5- and 7-years, respectively, *P* = 0.2). At all follow-up visits, more than 95% of the eyes achieved a CDVA of 20/25 or better and more than 85% a CDVA of 20/20. At the end of the follow-up (7 years), no eye lost more lines of CDVA, 56 eyes (66.7%) and 28 eyes (33.3%) gained lines of CDVA. At 7-years, the spherical equivalent was − 0.62 ± 0.62 D. No significant increase in IOP (> 20 mmHg or an increase higher than 5 mmHg) occurred in any case throughout the 7-year of follow-up. The loss in ECD from the preoperative baseline at the last follow-up visit was 2.6%. No intraoperative or postoperative complications or adverse events occurred during the follow-up period.

**Conclusions:**

The outcomes of this study show the long-term viability of the V4c ICL implantation as a surgical option for the correction of myopia.

## Background

The Visian Implantable Collamer Lens (ICL) (Staar Surgical AG, Nidau, Switzerland) with a central port design (V4c), has been widely accepted as effective and safe treatment for myopia correction [1]. The central port design (named KS-Aquaport; as a tribute to his inventor; Kimiya Shimizu) allows the circulation of the aqueous fluid through the lens [2, 3]. The lens was implanted first in 2007, since then, several studies with small and large samples have shown their results at different follow-ups supporting the use of this lens for the correction of moderate to high myopia [1].

Compared to the previous ICL models, the incidence of reported complications has significantly decreased with the V4c model [1, 4]. For example, the reported incidence of cataracts, which is the most common and significant complication with the previous model [4], has been reduced to almost 0 with the V4c model [1, 5], even for those cases with a low vault [6]. These findings support the idea that aqueous humour flow through the central hole of the lens maintains the normal physiology of the anterior segment of the eye, preventing potential complications. However, it should be noted there have been only a few long-term studies (spanning more than 3 years) [6–11] on the clinical outcomes of the V4c ICL implantation, and only three studies reached 5-years follow-up [6–8]. In contrast, several of the long-term studies (spanning more than 5 years) of the previous ICL design have been published [12–19]. Cumulative numbers of complications of ICL implantation are expected to increase with time [12, 16, 17, 20]. Furthermore, Yang et al. [11] recently, in a 4-year prospective study, found that the average lens density increased at 4 years after ICL V4c implantation, although no cataracts were reported during follow-up. It should be confirmed whether, over a longer follow-up, those changes in lens density will lead to a clinically significant cataract.

Although the ICL V4c implantation has shown to provide outstanding and stable visual and refractive outcomes and low adverse event rates, the follow-up periods in most of the studies ranged between 1 to 3 years [1], and there is a paucity of long-term studies of this new ICL model. This study aims to evaluate long-term clinical outcomes of the ICL V4c implantation for moderate to high myopia throughout a 7-year follow-up period.

## Methods

This retrospective, observational study comprised 84 eyes of 52 patients who underwent implantation of the Visian Implantable Collamer Lens (ICL, model V4c, STAAR Surgical Inc) to correct myopia at the Fernández-Vega Ophthalmological Institute, Oviedo, Spain, from January to December 2012. All patients provided written informed consent after the nature and possible consequences of the study were explained fully in accordance with the Declaration of Helsinki. The inclusion criteria were stable refraction with a myopic error in the range correctable with the V4c ICL (from − 1.00 D to − 18.00 D of sphere), a clear central cornea, anterior chamber depth (ACD) greater than 2.8 mm measured from the corneal endothelium to the anterior lens capsule, endothelial cell density (ECD) greater than 2000 cells/ mm^2^, mesopic pupil smaller than 7.0 mm, trabecular-iris angle (TIA) greater than 35° (grade III by gonioscopy), crystalline lens rise (CLD) less than 500 μm and postoperative follow-up period of at least 7 years. The exclusion criteria were cataract, history of glaucoma or retinal detachment, macular degeneration or retinopathy, neuro-ophthalmic disease, or any ocular inflammation history.

Before the surgery, patients had a complete ophthalmologic examination, including uncorrected distance visual acuity (UDVA), corrected distance visual acuity (CDVA), manifest and cycloplegic refractions, slit lamp examination, keratometry, corneal topography, pachymetry and white-to-white (Sirius, CSO Ophthalmic, Italy), ACD and angle to angle (OCT Visante, Carl Zeiss Meditec, Germany &), ECD measurement (SP 3000P, Topcon Europe Medical), intraocular pressure (IOP) measurement by Goldmann applanation tonometry, and anterior segment optical coherence tomography (OCT; Visante, Carl Zeiss Meditec AG).

All included eyes in this study had implantation of the myopic V4c Visian ICL model. The details of the lens have been published previously [8]. Emmetropia was selected as the postoperative target refraction for all eyes. ICL power calculation was performed using a modified vertex formula provided by the manufacturer (Staar Surgical). ICL size was individually determined based on the horizontal white-to-white (WTW) distance, ACD measured by Scheimpflug photography, and angle-to-angle distance measured with OCT. To prevent a postoperative vault greater than 1000 μm, the following protocol, based on the surgeon’s experience, was applied: In cases where the distance from the ICL to the angle-to-angle diameter (ATA) was higher than 800 μm, or had a pupillary ovalization or compromised pupillary dynamic in the postoperative visit of the first day, we verified that the vertical angle-angle was longer than the horizontal, and subsequently, the ICL was rotated 90° to vertical orientation.

The same experienced surgeon (JFA) performed all surgeries following the standard procedure previously described [8, 9, 21]. Postoperative follow-up visits were scheduled at 1-day, 1 week and at 1, 3 and 12 months and then every 1 year thereafter. The analysis included the outcomes from preoperative, and 1-, 5-, and 7-years visits. The examinations included measurement of UDVA and CDVA, manifest refraction, slit-lamp examination, IOP, ECD and fundoscopy. The central distance between the ICL and the crystalline lens (vault) was assessed using OCT. The vault between the crystalline lens and the ICL was measured perpendicular to the lens apex or at the narrowest point.

Data analysis was performed using SPSS for Windows, version 14.0 (SPSS Inc., Chicago, IL). Normality was checked with the Kolmogorov-Smirnov test. One-way repeated-measures analysis of variance (ANOVA) with a Bonferroni post-hoc test was performed to compare results. Differences were considered to be statistically significant when the *P* value was less than 0.05.

## Results

This study included 84 eyes of 52 patients (17 men and 35 women). All patients completed the follow-up period of 7 years and attended all the follow-up visits. Table 1 summarizes preoperative demographic data of the patients and ICL characteristics. The distribution of the lens sizes implanted were: 13.7 mm in 9 eyes (10.7%), 13.2 mm in 53 eyes (63.1%), and 12.6 mm in 22 eyes (26.2%).

**Table 1:** Preoperative patient demographics and ICL characteristics

	Mean ± SD	Range [Min, Max]
Age (years)	31.04 ± 4.89	[25, 50]
Refraction sphere (D)	−9.02 ± 2.85	[−17.50, −4.0]
Refraction cylinder (D)	−0.65 ± 0.51	[−1.5, 0]
Spherical Equivalent (D)	−9.35 ± 2.90.60	[−18.25, −4.50]
UDVA (logMAR)	1.63 ± 0.38	[0.7, 2.0]
CDVA (logMAR)	0.04 ± 0.12	[0.0, 0.4]
Minimum Keratometry (D)	43.26 ± 1.64	[40.00, 46.00]
Maximum Keratometry (D)	44.18 ± 1.69	[40.78, 47.00]
Corneal thickness (μm)	530 ± 37	[448, 630]
ACD (mm)	3.13 ± 0.22	[2.80, 3.75]
WTW (mm)	11.95 ± 0.47	[10.99, 13.45]
ATA (mm)	11.74 ± 0.37	[11.00, 13.20]
ECD (cells/ mm^2^)	2640 ± 336	[2000, 3903]
IOP (mmHg)	13.02 ± 1.78	[8, 19]
ICL sphere (D)	−10.17 ± 2.70	[−18.0, −5.0]
ICL size (mm)	13.10 ± 0.33	[12.6, 13.7]

*ICL* implantable collamer lens, *D* dioptres, *UDVA* uncorrected distance visual acuity, *CDVA* corrected distance visual acuity, *ACD* anterior chamber depth, *WTW* white to white, *ATA* angle to angle, *ECD* endothelial cell density, *IOP* intraocular pressure, *SD* standard deviation

### Effectiveness and safety outcomes

**Fig. 1:**
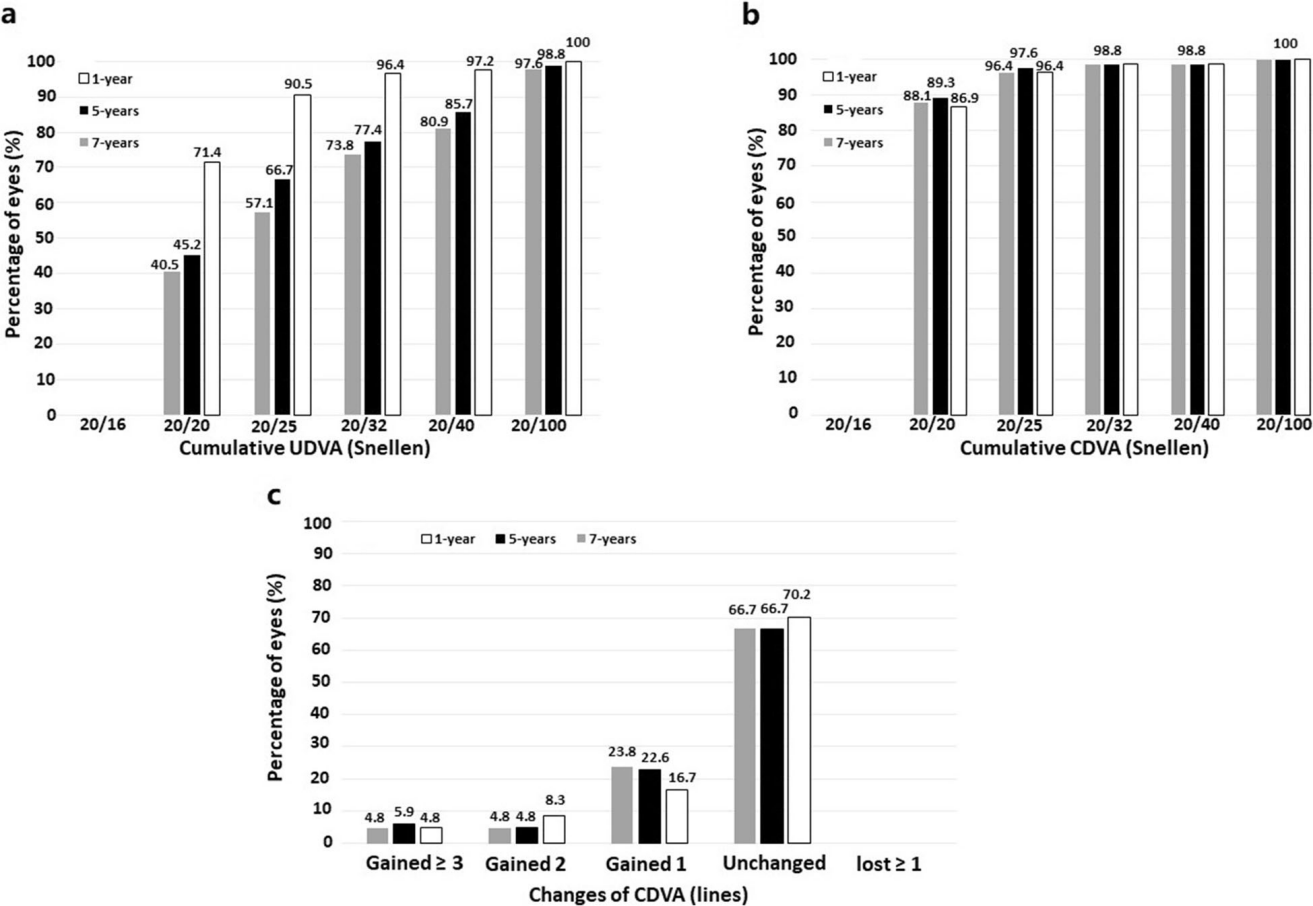
Cumulative uncorrected distance visual acuity (UDVA) (**a**) and corrected distance visual acuity (CDVA) (**b**) at 1-, 5- and 7-years post-surgery. **c** Variation in CDVA between preoperative and each postoperative follow-up visits

The mean postoperative UDVA (logMAR) was 0.04 ± 0.11, 0.13 ± 0.19 and 0.17 ± 0.23 at 1-, 5- and 7-years, respectively (*P* < 0.0001). The efficacy index (mean postoperative UDVA/mean preoperative CDVA) was 1.01, 0.85 and 0.80 at 1-, 5- and 7-years after surgery, respectively. Figure 1a shows the cumulative UDVA at each follow-up visit. The mean CDVA increased from the preoperative 0.04 ± 0.12 logMAR to 0.01 ± 0.06 logMAR at 1-year after surgery (*P* < 0.0001) and remained unchanged throughout a 7-year follow-up period (0.02 ± 0.08 logMAR and 0.02 ± 0.08 logMAR, at 5- and 7-years, respectively, *P* = 0.2). At all follow-up visits, more than 95% of the eyes achieved a CDVA of 20/25 or better and more than 85% a CDVA of 20/20 (Fig. 1b). Figure 1c shows the changes in CDVA between preoperative and each postoperative follow-up visits. At the end of the follow-up (7 years), no eye lost more lines of CDVA, 56 eyes (66.7%) did not change from preoperative, 20 eyes (23.81%) gained 1 line, 4 eyes (4.76%) gained 2 lines, and 4 eyes (4.76%) gained more than 2 lines of CDVA. The safety index (ratio between the postoperative CDVA and the preoperative CDVA) was 1.05 throughout the 7-year follow-up period.

### Predictability and stability

**Fig. 2:**
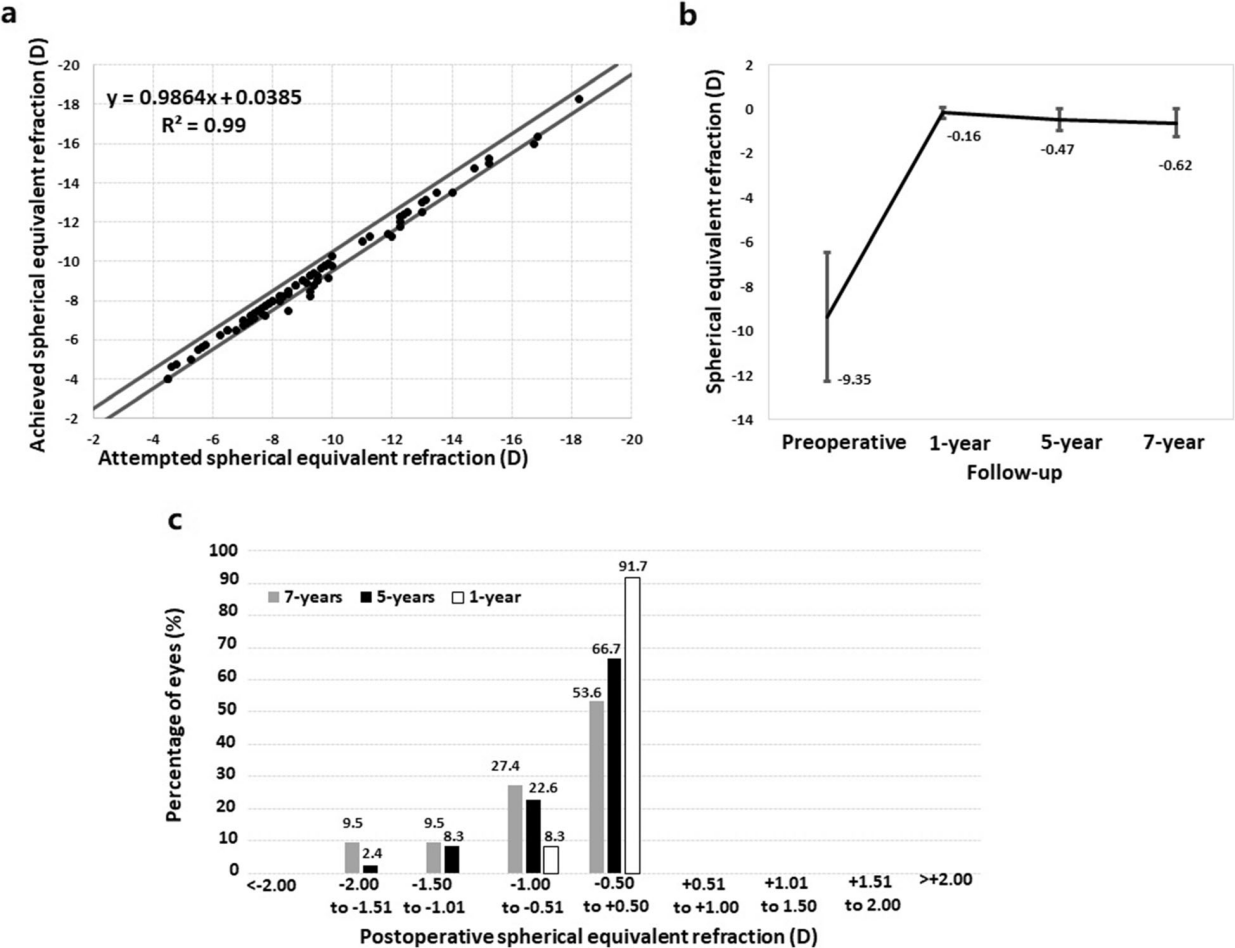
Plot of attempted versus achieved spherical equivalent at 1-year post surgery (predictability) (**a**) Time course (**b**) and accuracy (**c**) of manifest spherical equivalent over the follow-up

Figure 2a shows a scatterplot of the attempted versus achieved spherical equivalent refraction at 1-year post-surgery. Seventy-seven eyes (91.7%) were within ±0.50 D of the desired refraction (emmetropia) and all eyes (100%) were within ±1.00 D. The change in manifest spherical equivalent is shown in Fig. 2b. At 1-, 5- and 7-years after surgery, the spherical equivalent was − 0.16 ± 0.26 D, − 0.47 ± 0.48 D, and − 0.62 ± 0.62 D, respectively. Multiple comparisons showed statistically significant differences among all postoperative visits (Fig. 2b; *P* = 0.0006). At 5- and 7- years, 66.67 and 53.57% of the eyes, respectively, were within ± 0.50 D of the desired refraction; while 89.29 and 80.95%, respectively, were within ± 1.00 D (Fig. 2c).

### Intraocular pressure, endothelial cell density, and vault

**Fig. 3:**
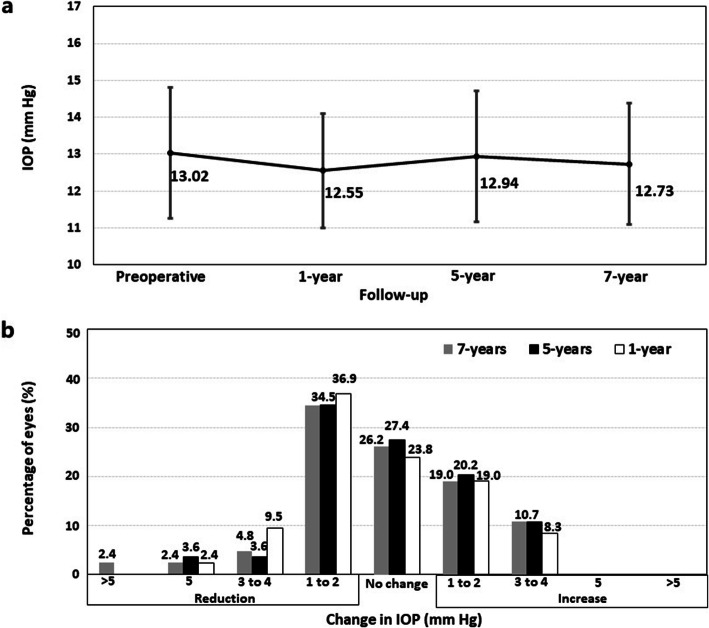
The time course of the mean IOP over the whole follow-up period (**a**) and variation in intraocular pressure (IOP) between preoperative and each postoperative follow-up visits (**b**)

Figure 3a shows the time course of the mean IOP over the follow-up period. A slight statistically significant reduction was found between preoperatively and 1 year after surgery (13.02 ± 1.77 mmHg and 12.54 ± 1.53 mmHg, respectively, *P* = 0.02). Subsequently, the mean IOP remained stable over the 7-years of follow-up (12.9 ± 1.77 mmHg and 12.69 ± 1.64 mmHg at 5- and 7-years, respectively, *P* = 0.07). Figure 3b shows the changes in IOP between preoperative and each postoperative follow-up visits. At the end of the follow-up, the largest proportion of the eyes showed a reduction in IOP (37 eyes, 44.1%), in 22 eyes (26.2%) the IOP did not change from the preoperative value, 16 eyes (19.0%) experienced an increased 1–2 mmHg, and 9 eyes (10.7%) had an increased 3–4 mmHg. No significant increase in IOP (> 20 mmHg or an increase higher than 5 mmHg) occurred in any case throughout the 7-year of follow-up.

**Fig. 4:**
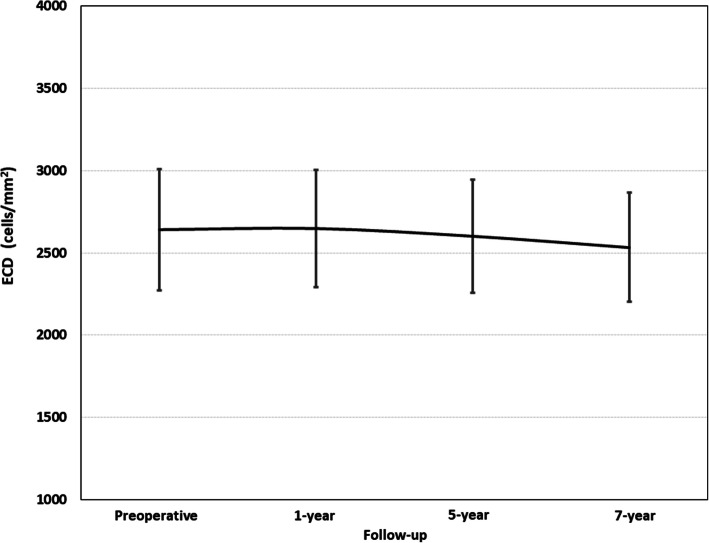
Change in mean endothelial cell density (ECD) (cells/mm^2^) throughout the entire follow-up period

Figure 4 shows the ECD over the follow-up. There were no significant changes in the mean ECD at any timepoint (*P* = 0.07). The loss in ECD from the preoperative baseline compared with the last follow-up visit was 2.6%.

**Fig. 5:**
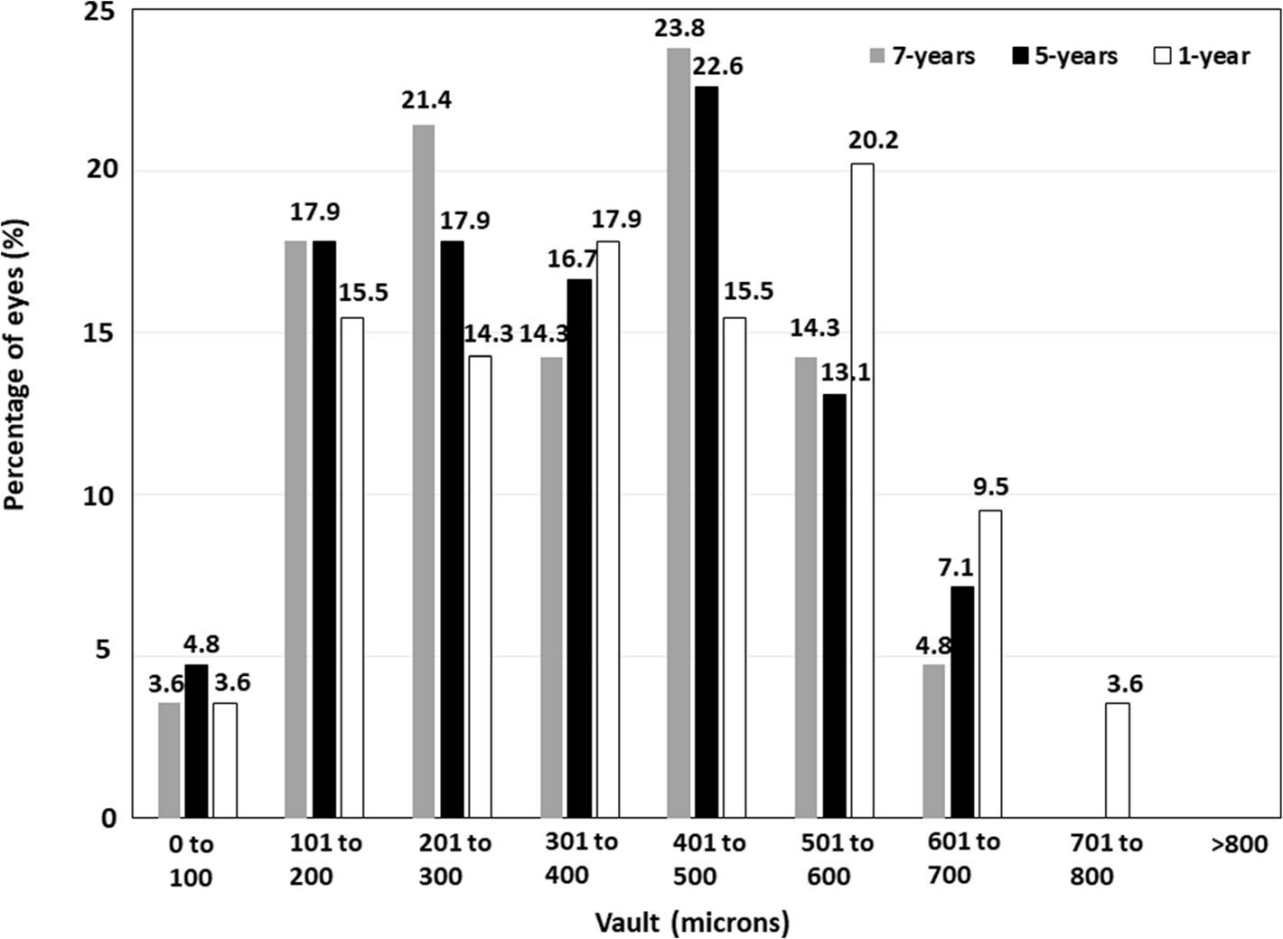
Distribution of eyes according to the vault, measured in microns, at 1-, 5- and 7-years post-surgery

The mean postoperative vault was reduced from 400 ± 180 μm at 1 year to 355 ± 160 μm at 5 years postoperatively (*P* < 0.0001). Subsequently, it remained stable from 5 to 7 years after surgery (348 ± 150 μm at 7 years; *P* = 0.07). Figure 5 shows the postoperative distribution of vault. No eyes showed a vault higher than 800 μm at any timepoint. Around 20% of eyes had a vault lower than 200 μm throughout the whole follow-up.

### Adverse events and secondary surgeries

There were no intraoperative complications, and no eye required ICL explantation or exchange. Over the whole follow-up period, no cases of anterior subcapsular opacity, cataract, pigment dispersion glaucoma, pupillary block, or other vision-threatening complications were reported. Two eyes of the same patient required laser touch up to correct residual refractive error 2 years after ICL implantation. In 4 eyes (4.76%), the ICL was rotated 90° to vertical orientation due to a high vault at the 1-day postoperative visit. In the four eyes, the postoperative vault dropped below 500 μm, and the visual and refractive outcomes were stable over the entire follow-up. At the end of the follow-up, the IOP change from preoperative ranged − 1 to + 1 mmHg, and the ECD loss ranged between 1.9 and 2.9%. In summary, the long-term results in these 4 eyes were similar to the whole group where ICL was implanted horizontally.

## Discussion

The present study reports the outcomes throughout 7 years of follow-up in patients implanted with the V4c ICL model. It aimed to evaluate whether the outstanding visual and refractive results, and low adverse event rates previously reported with the V4c ICL model in shorter-follow-up studies will persist over a longer follow-up period.

During the first 5 years of follow-up, the safety index was similar to those previously reported at various postoperative intervals [1], remaining stable also up to the 7-year follow-up period (1.05). At all follow-up visits, more than 95% of the eyes achieved a CDVA of 20/25 or better and more than 85% a CDVA of 20/20. These results support the excellent stability of the CDVA outcomes, and thus confirm the safety of the procedure. The efficacy index at 1 year was 1.01, this agrees with those previously found, that show short-term efficacy indices of 1.00 or greater. This result implies that the postoperative UDVA is equal to or better than preoperative CDVA. However, longitudinal studies show that this index worsens slightly over time [8, 9]. The current study confirms this trend in a longer follow-up since the efficacy index at 5 years was worse than at 1 year, and in turn, at 7 years, it worsened compared to 5 years (1.01, 0.85 and 0.80 at 1-, 5- and 7-years after surgery, respectively). It is important to note that the efficacy index is based on postoperative UDVA. Hence, it would be directly affected by a change in the refractive error over time. As will be discussed below, our study showed an increase in the mean spherical equivalent of around − 0.50 D between 1 and 7 years after surgery. Igarashi et al. [13] found the increase in axial length is the main factor for myopia progression after ICL implantation. Although the causes for this slight myopia progression could be multifactorial, the excellent outcomes of CDVA in terms of stability make it plausible to think that it was related to a continuous axial elongation and not to any other source associated with the ICL implantation procedure. Hence, this could affect any other refractive procedure.

Our refractive outcomes also confirmed satisfactory predictability results. At 1 year, 91.7 and 100% of eyes were within ± 0.50 D and ± 1.00 D of emmetropia, respectively. This finding agrees with those previously reported since almost all the studies reported that 100% of ± 1.00 D over their postoperative periods [7–10, 22–25]. This accuracy rate slightly decreased over time [7–9]. At 5- and 7- years, 90.2 and 82.4%, respectively, were within ± 1.00 D (Fig. 2c). As previously indicated, these changes may be due to an axial elongation occurring over time. However, it should be noted that the mean spherical equivalent preoperatively was − 9.35 D and at 7-years after surgery was − 0.62 D while the change in the mean spherical equivalent throughout the 7 years was around − 0.5 D. Hence, these values confirm the excellent refractive results of this procedure. Furthermore, for those cases that residual myopia provides an unsatisfactory UDVA level, a laser touch-up can be effectively and safely planned to correct residual refractive error after ICL implantation.

Although all previous studies had a shorter follow-up (up to 5 years), the satisfactory visual and refractive results previously reported were expected to remain throughout the 7 years of the follow-up period of our study. However, beyond the visual and refractive outcomes, studies spanning more than 5 years are crucial for evaluating physiological changes and potential adverse events associated with any intraocular procedure. The rate of adverse events with the previous ICL models (such as cataracts, ECD loss, pigment dispersion syndrome) were showed to increase with time [12, 16, 17, 20].

Guber et al. [12] reported that the rate of lens opacity development increased around 15% between 5 and 7 years after ICL V4 implantation. After 8 years of ICL V4 implantation, the incidence of cataract formation reported by Igarashi et al. [13] was higher than that reported in studies with a shorter follow-up. Choi et al. [14] showed that lens opacity developed after ICL V4 implantation occurred at a mean of 7.3 ± 2.2 years postoperatively. Nakamura et al. [16] reported that 10.5% of the eyes developed an anterior cataract during the 5- to 10-year follow-up period. The prevalence of cataracts after ICL V4c implantation has been reported to be around 0.17% [1], however, the longest follow-up studies published to date only reached a maximum of 5 years follow-up [6–8]. In this study, extending the follow-up to 7 years, we did not find cataract formation in any cases. One of the main risk factors associated with developing anterior capsular cataract development with the older ICL models was a low vault. Fernandes et al. [4], in a review of the potential complications of the previous models of ICL, reported that in 33.8% of ICL-induced cataracts, the vault was lower than 200 μm. In this study, around 20% of the eyes had a vault lower than 200 μm throughout the follow-up. These findings corroborate that the central hole of the V4c ICL model prevents cataract development, even in eyes with a low vault.

Regarding IOP, the central hole offers surgical advantages over non-hole ICL models since no preoperative iridotomy or intraoperative iridectomy is necessary to prevent IOP increase related to pupillary block or chronic pigment dispersion [4]. Shimizu et al. [7] showed comparative IOP values between eyes implanted with a hole-equipped ICL and a non-hole ICL, over a follow-up period of 5 years. Furthermore, previous studies with short-, medium- and long-term follow-up showed that there no significant variation of IOP over time [1] after ICL V4c implantation. However, it is important to be cautious because the longest follow-up to date was 5 years. In the Guber et al. [12] study, around 13% of the cases developed ocular hypertension at a mean of 7.3 years after ICL V4 implantation. In our study, the mean IOP remained stable over the 7-years of follow-up (Fig. 3a). At 7 years of the surgery, the largest proportion of the eyes showed a reduction in IOP from the preoperative value, and no significant increase in IOP (> 20 mmHg or an increase higher than 5 mmHg) occurred in any case throughout the 7-year of follow-up (Fig. 3b). These results suggest that central hole prevent the IOP increase that may be associated with pupillary block or chronic pigment dispersion [4]. In addition to the central hole ICL design, it should be noted that our study showed no eyes with a vault higher than 800 μm at any timepoint. To prevent a postoperative high vault, we rotated the ICL 90° to vertical orientation in four eyes so that the distance between the ICL and the ATA was higher than 800 μm in the postoperative visit of the first day. In all 4 eyes, the postoperative vault dropped below 500 μm after ICL vertical rotation, all the parameters studied were stable over the entire follow-up, and no associated complications were found in these four eyes. The sulcus has a vertically oval shape, with the vertical diameter longer than the horizontal one [26, 27]. Consequently, it is expected that the ICL rotation to a vertical alignment should reduce the vault, and thus the postoperative complications related to a high vault, without the need of an ICL exchange for a smaller size one. Of note, before performing this surgical manoeuvre, it is mandatory to confirm that the vertical diameter is longer than the horizontal one. Currently, intraoperative OCT allows us to measure the vault intraoperatively, hence, for those cases with an extreme intraoperative vault, the ICL can be rotated during the same surgical session. The intraoperative OCT represent an outstanding tool to prevent potential complications related to an inadequate vault. It would be interesting to carry out future studies to establish intraoperative vault values safety cut-off.

Finally, our results did not reveal a statistically significant change in ECD over the 7 years of follow-up. The loss in ECD from the preoperative baseline compared to the last follow-up visit was 2.6%. This is in good agreement with those previously reported, suggesting that the ICL does not induce a significant ECD loss over long periods [1]. However, it is interesting to note that Yang et al. [10] found that excessively high vault values increased the risk of ECD loss. Hence, the vertical ICL rotation manoeuvre performed in 4 eyes (4.76%) may have avoided a risk factor (high vault) for a potential ECD loss over time.

## Conclusion

The outcomes of the present study indicate that the use of the V4c ICL model for the correction of myopia was overall satisfactory in terms of effectiveness, safety and stability during 7-years of follow-up, which shows its long-term viability as a surgical option for the correction of myopia.

## Data Availability

Not applicable

## Abbreviations

ICL: Implantable Collamer Lens
UDVA: Uncorrected distance visual acuity
CDVA: Uncorrected distance visual acuity
IOP: Intraocular pressure
ECD: Endothelial cell density
ACD: Anterior chamber depth
TIA: Trabecular-iris angle
CLD: Crystalline lens rise
OCT: Optical coherence tomography
WTW: White-to-white
ATA: Angle-to-angle diameter
